# Lesch-Nyhan syndrome a dental approach: case report

**DOI:** 10.4314/ahs.v25i2.37

**Published:** 2025-06

**Authors:** Priscilla Sena Souza Luz Campos, Luís Cândido Pinto da Silva, Paulo Franco Taitson, Lara Alves da Silva Oliveira, Laura Camilo Bautista

**Affiliations:** 1 Department of Pediatric Dentistry, Pontifical Catholic University of Minas Gerais, Belo Horizonte, MG, Brazil; 2 São Francisco de Assis Hospital, Belo Horizonte, MG, Brazil

**Keywords:** Lesch-Nyhan Syndrome, HGPRT Deficiency, Self-mutilation, Lip Bumper

## Abstract

Lesch-Nyhan syndrome was first described in 1964. It is an X-linked recessive disorder caused by the absence of the enzyme hypoxanthine-guanine phosphoribosyl ransferase (HGPRT), which is involved in the metabolism of purines (nitrogenous bases that make up nucleotide). The absence of the enzyme leads to motor and neurological dysfunction, increased uric acid production, and mental retardation. One of the main characteristics of this syndrome is compulsive self-mutilation, such as biting the lips, tongue, and fingers. Men are the most affected and the prevalence is 1/380,000 live births. Several types of treatments are managed to reduce the manifestations of Lesch-Nyhan Syndrome, such as the placement of oral appliances, dental extractions, and drug therapy, the main one being Allopurinol, which can reduce the concentration of uric acid in the body. The role of the dentist becomes essential since teeth represent the main instrument for self-mutilation. In this sense, the article aims to describe the Lesch-Nyhan syndrome, the possible treatments and report a clinical case of a 4-year-old male patient who presented the syndrome.

## Literature review

In 1964, doctors Michael Lesch and Bill Nyhan reported a new syndrome, which was named Lesch-Nyhan Syndrome. This, in turn, was found in two brothers, who had motor and neurological disorders, hyperuricemia, and self-mutilation. In 1967, the physician Seegmiller linked the above syndrome to a deficiency of the enzyme HGPRT (hypoxanthine-guanine phosphoribosyltransferase), which increases the production of uric acid in the body^1^. Lesch-Nyhan syndrome is an inherited condition that mostly affects men. It is characterized by a total or partial mutation of the HGPRT gene, causing a malformation of the enzyme, which, as it does not perform its function, increases the level of uric acid, causing several diseases, such as nephrotiasis, hyperuricemia, gout, renal dysfunction, uricosuria, motor and neurological dysfunction, and self-mutilation^2^. Symptoms usually occur between 3 and 6 months of life, a period in which there is a delay in nervous system involvement, making it difficult to raise the head and sit down. Self-mutilation presents itself when the child's teeth begin to erupt. And because of this, the child starts to bite lips, thus advancing to fingers and hands.

Allopurinol is one of the drugs indicated for patients with the syndrome, since it can reduce the concentration of uric acid, thus preventing the development of kidney damage, thus delaying kidney failure. Several other types of treatments can be approached to reduce the manifestations of Lesch-Nyhan Syndrome, such as drug therapy (benzodiazepines, neuroleptics, antidepressants, and anticonvulsants) which is used as an intervention and preventive establishment, placement of oral appliances, dental extractions and orthognathic surgeries^3^. The aim of this article is to report a clinical case of a 4-year-old male patient who attended the Clinic for Special Patients of the School of Dentistry, of Pontifical Catholic University of Minas Gerais (PUC-MG), Brazil. The person responsible for the patient came to the clinic with the goal of finding other ways to control self-mutilation and to improve neurological conditions, since self-mutilation caused great tissue loss in the region of the lower lip, tongue and cheeks. A Lip Bumper was then made and placed to protect the lower lip against self-mutilation.

## Case Report

**Photo 1:**
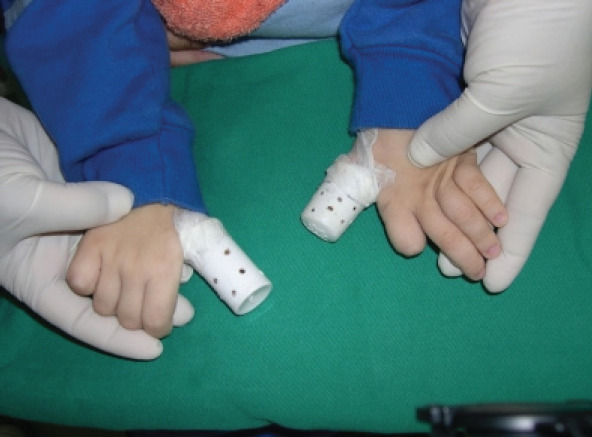
Protective devices made with polyvinyl chloride (PVC) tubing to prevent self-mutilation

Patient H.A.S., male, 4 years old, leukoderma, after showing self-mutilating behavior, with great tissue loss in the lower lip, tongue and cheeks, was referred for treatment at the Clinic for Special Patients of the School of Dentistry of Pontifical Catholic University of Minas Gerais (PUC-MG), Brazil. The mother reports that the child was born without complications during delivery. However, during the neonatal period, he had reflux, but the mother could not say whether it was gastroesophageal or vesicourethral. At three months of age, the patient started presenting behavioral disorders with delayed psychomotor development and, in addition, a urinary tract infection was detected, which was treated in seven days of hospitalization. At 1 year and 9 months old, the child developed recurrent urinary tract infection and was treated with nitrofurantoin, being monitored by the nephrology team. An elevated uric acid/creatinine ratio is a good indicator of excess uric acid. In children under ten years of age affected by Lesch-Nyhan Syndrome, a Uric acid/Creatinine ratio greater than 2 is a typical finding. The child in question was undergoing symptomatic treatment of signs and symptoms of Lesch-Nyhan syndrome at 1 year and 9 months. The diagnosis of Lesch-Nyhan Syndrome was made at the Hospital Das Clinicas of Federal University of Minas Gerais (UFMG – Brazil) through laboratory tests (uric acid)/creatinine ratio) that reached level 4. The characteristics of self mutilation, neurological deficit and spasticity completed the medical finding. As it is essential that excessive uric acid production be controlled, to reduce the risk of nephropathy and nephrolithiasis, occasional ingestions of allopurinol have been reported. Spasticity was also reduced with occasional use of benzodiazepines. Self-injury and other behaviors were attempted to be controlled using mechanical restraint. The neurological and psychiatric manifestation of self-mutilation began at 2 years of age, when multiple lesions were observed on the right and left thumb. In view of this, protective devices were made using Polyvinyl Chloride (PVC) tubes (Photo 1).

**Photo 2:**
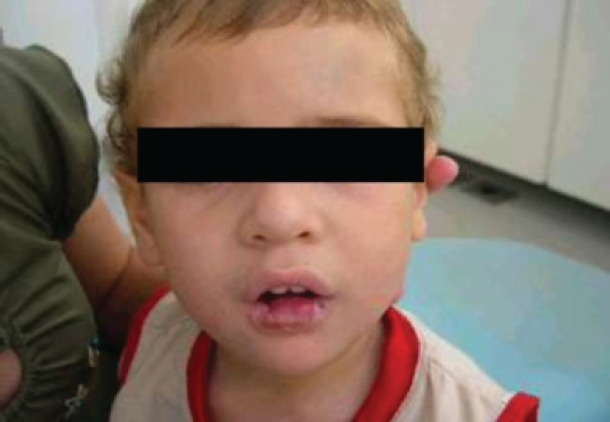
Patient at his first appointment at the Clinic for Special Patients of the School of Dentistry of Pontifical Catholic University of Minas Gerais (PUC-MG), Brazil

**Photo 3:**
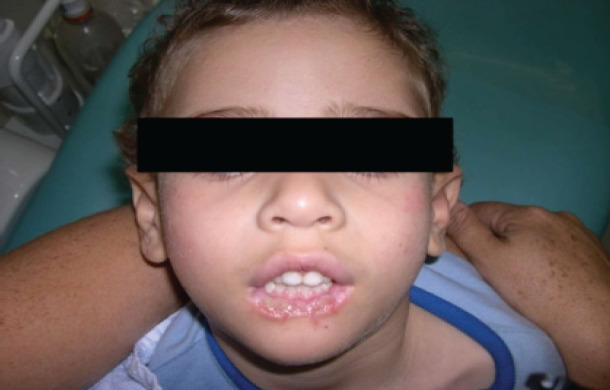
Lower lips with great tissue loss

**Photo 4:**
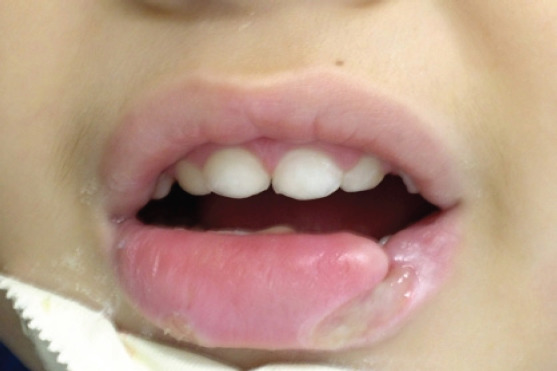
Lower lips with great tissue loss

The child, through the biting process, began to mutilate his lower lip, thus finding signs of mutilation on his cheeks and tongue, since his thumbs were already protected (Photos 2,3 and 4). Given the clinical picture, the patient was referred to the General Pediatric Center (GPC) and, later, to the PUC-MG School of Dentistry to try to improve the neurological conditions and, at the same time, control the self-destructive manifestations. The initial assessment on the GPC revealed a child with clear cognitive impairment. The clinical oral examination showed no caries activity, good hygienic conditions, which indicated that he was well cared by his guardians. Due to the severe compromise of oral structures, less invasive alternatives were considered, since the extractions indicated in the literature are more radical. It was then indicated the preparation and placement of an active lip plate (Lip Bumper), which is characterized by keeping the lower lips away from the teeth, preventing the patient from damaging them. This procedure would be performed in a hospital under general anesthesia. Unfortunately, due to successive pulmonary complications, resulting in several hospitalizations, the patient died, and it was not possible to complete his oral rehabilitation.

## Discussion

Lesch-Nyhan syndrome was first described in 1964, when physicians Michael Lesch and Willian Nyhan detailed a case of two brothers with choreoathetosis, mental retardation, and self-mutilation, associated with a metabolic abnormality in the biosynthesis of purines (adenine and guanine). Its prevalence is estimated at 1/380,000 live births^4^. The syndrome is a rare genetic disorder of recessive inheritance associated with a mutation of the HGPRT gene, located on the X chromosome; this gene is responsible for encoding the enzyme hypoxanthine-guanine phosphoribosyltransferase. Men are the most affected, and heterozygous women are carriers, but they are usually asymptomatic, as it is an X-linked disease^5^. Babies affected by the disease develop normally during the 3 to 6 months of life. The first sign of the disease is usually the appearance of what looks like orange sand in the diapers or crystalluria with obstruction of the urinary tract and, in the first year of life, a psychomotor delay in logical development becomes evident and the child is unable to sit up by himself or loses this ability, in addition to presenting mental retardation^6^.

Deficiency or lack of HGPRT leads to increased concentration of uric acid, triggering several diseases such as nephrothiasis (kidney stones), hyperuricemia (high levels of uric acid in the blood), gout, kidney dysfunction, uricosuria (uric acid in the urine), crystal formation in the joints, motor and neurological dysfunction such as occasional choreoathetosis (involuntary movements) or spasticity, dysarthria (los of the ability to articulate words normally) and dysphagia (difficulty swallowing food or liquids) as well as self-mutilation of the lips, tongue and fingers^4^-^6^. Compulsive self-injurious behavior is one of the hallmark features of Lesch-Nyhan syndrome; patients begin to bite their lips, tongue or fingers, leading to tissue loss and partial amputations. Self-mutilation has recently been attributed to an obsessive-compulsive behavior, usually relieved when the patient protected from oneself. In some cases, the aggressive behavior is also directed against family and friends, with patients spitting or using abusive language^7^. The excessive production of uric acid can be controlled with Allopurinol, a drug used by the patient reported in this article, which blocks the conversion of xanthine and hypoxanthine into uric acid. With this drug, uric acid and urate levels in the urine decrease, avoiding crystalluria, nephrolithiasis, and gouty arthritis. Although, Allopurinol has no effect on the neurological part of the syndrome, it does not modify the self-mutilation behavior^8^-^9^.

The use of Botulinum Toxin A (BTX-A) injected bilaterally into the masseter muscles temporarily prevents synaptic pre-release of acetylcholine, causing muscle dysfunction and weakness. This treatment results in a reduction in self-mutilation behavior. In addition to botulinum toxin, benzodiazepines, neuroleptics, antidepressants, chloral hydrates, and anticonvulsant drugs have been sought for inhibition of this compulsive behavior. Recent therapeutic trials also include gabapentin, which is free of side effects acting on the central and peripheral nervous system, and dopamine replacement therapy and deep brain stimulation in the globus pallidus^10^.

The literature shows that in some patients, extracting part or all of the deciduous teeth can be one of the effective ways to face self-mutilation, in which case tooth extraction is also necessary in the permanent dentition, since it reduces soft tissue damage, but it is an extremely invasive approach and, in certain post-extraction cases, it generated a significant deformity in the patient's mouth and perioral tissues^3^,^11^. This would not be an approach used in the case described. Our planning involved a more conservative approach and subsequent follow-up of the case, considering the less invasive treatments indicated in the literature, such as the use of intra-oral protective devices, which have been used as the first measure to avoid oral self-mutilation, including a variety of devices, such as protector's labial and lingual, with variable success^3^,^9^.

Arhakis et al., reported in their paper, a posterior bite plate proposed to prevent traumatic injuries to the tongue and lips to improve the oral problems in a 14-year-old boy. His approach was very well accepted by the parents, who were not willing to accept extraction of anterior teeth as a treatment option. During the 3-year follow-up period, the signs of self-mutilation disappeared^3^. The authors' findings corroborate the success achieved in our treatment, which aimed to prevent the trauma caused by the self-mutilation of the described patient. In Gaslini Hospital, together with its orthodontic team, created a new resin orthodontic device to preserve the oral and perioral tissue of patients with Lesch-Nyhan syndrome. This device is a mouthguard with two different matrices: hard and soft. The hard matrix ensures good retention in the oral cavity, while the soft matrix protects the oral and perioral tissue from traumatic biting. The effectiveness of this device was observed over a period of about 7 years, during these years there were no self-inflicted oral and/or perioral mutilations. The main difficulties encountered during this therapy are the need to modify the device to adapt it to oral changes and the risk of small cracks in the resin occlusal bite that must be repaired frequently^11^. For this reason, we would opt for the installation of the Lip Bumper device. In another recent case report, a 31-week-old patient with the syndrome and signs of self-mutilation in oral tissues was reported. A fixed-type lip protector was planned and executed to prevent biting habits; however, the patient did not adapt, and a semi-fixed lip protector was made for the upper and lower arches, with follow-up a recovery of the injured tissues was noticed, and no new injury was observed^12^. In our case, it was not possible to continue the patient's oral treatment. Regardless of our outcome, this article aims to describe and bring knowledge about Lesch-Nyhan Syndrome and the devices used and recommended, analyzing the role of the dental surgeon for dental treatment and the improvement in the quality of life of patients with this syndrome. The literature shows several treatment approaches for mutilation. Although we did not have a long follow-up of our case, the use of the Lip Bumper would be our first choice, because it brings positive results and is not an extreme measure, such as extraction of all deciduous or permanent teeth.

## Conclusions

A multidisciplinary follow-up in patients with Lesch-Nyhan Syndrome becomes essential, and the dentist is an important part of this team, since self-mutilation is one of the main characteristics of the syndrome, and the tool for mutilation is the teeth. This case highlights the complexity of the Lesch-Nyhan Syndrome and the care that we must take regarding to the child and family.
